# An *In Silico* Investigation to Explore Anti-Cancer Potential of *Foeniculum vulgare* Mill. Phytoconstituents for the Management of Human Breast Cancer

**DOI:** 10.3390/molecules27134077

**Published:** 2022-06-24

**Authors:** Baljinder Kaur, Rajan Rolta, Deeksha Salaria, Balvir Kumar, Olatomide A. Fadare, Renato Araujo da Costa, Ajaz Ahmad, Mahmood Basil A. Al-Rawi, Mohammad Raish, Irfan A. Rather

**Affiliations:** 1 Systems Biology Laboratory, Department of Biotechnology, Punjabi University Patiala, Patiala 147002, Punjab, India; 2 Faculty of Applied Sciences and Biotechnology, Shoolini University, Solan 173212, Himachal Pradesh, India; roltarajan612@gmail.com (R.R.); deekshasalaria20@gmail.com (D.S.); 3 University Institute of Biotechnology, Chandigarh University, Mohali 140413, Punjab, India; balvir.e10913@cumail.in; 4 Organic Chemistry Research Lab, Department of Chemistry, Obafemi Awolowo University, Ile-Ife 220282, Nigeria; tofadare@oauife.edu.ng; 5 Federal Institute of Education, Science, and Technology of Para, Belém 66000-000, Para, Brazil; renatoacifpa@gmail.com; 6 Department of Clinical Pharmacy, College of Pharmacy and King Saud University, P.O. Box 2457, Riyadh 11451, Saudi Arabia; ajukash@gmail.com; 7 Department of Optometry, College of Applied Medical Sciences, King Saud University, Riyadh 11451, Saudi Arabia; malrawi@ksu.edu.sa; 8 Department of Pharmaceutics, College of Pharmacy and King Saud University, P.O. Box 2457, Riyadh 11451, Saudi Arabia; mraish@ksu.edu.sa; 9 Department of Applied Microbiology and Biotechnology, Yeungnam University, Gyeongsan 38541, Korea; rather@ynu.ac.kr

**Keywords:** phytoconstituents, breast cancer, molecular docking, MD simulation

## Abstract

Breast cancer is one of the most prevalent cancers in the world. Traditionally, medicinal plants have been used to cure various types of diseases and disorders. Based on a literature survey, the current study was undertaken to explore the anticancer potential of *Foeniculum vulgare* Mill. phytoconstituents against breast cancer target protein (PDB ID: 6CHZ) by the molecular docking technique. Molecular docking was done using Autodock/vina software. Toxicity was predicted by the Protox II server and drug likeness was predicted by Molinspiration. 100 ns MD simulation of the best protein-ligand complexes were done using the Amber 18 tool. The present molecular docking investigation has revealed that among the 40 selected phytoconstituents of *F. vulgare*, α-pinene and D-limonene showed best binding energy (−6 and −5.9 kcal/mol respectively) with the breast cancer target. α-Pinene and D-limonene followed all the parameters of toxicity, and 100 ns MD simulations of α-pinene and D-limonene complexes with 6CHZ were found to be stable. α-Pinene and D-limonene can be used as new therapeutic agents to cure breast cancer.

## 1. Introduction

*Foeniculum vulgare* Mill. is an important medicinal herb and one of the most widely cultivated spice plants that belongs to the family Umbelliferae. *Foeniculum vulgare* is a nutritionally rich reservoir of carbohydrates, proteins, monounsaturated and polyunsaturated fatty acids, short chain fatty acids (SCFA), minerals, vitamins, and energy. Previous scientific reports provide a comprehensive collection of the in vitro and in vivo pharmacological investigations which reflect the efficacy of various *Foeniculum vulgare* constituents in treating arthritis, cancers, conjunctivitis, digestive disorders, endocrine issues, hepatic, kidney, reproductive, and respiratory ailments due to their anti-inflammatory, anti-microbial, anti-mutagenic, anti-nociceptive, anti-oxidant, anti-spasmodic, anti-thrombotic, anti-tumor, anti-viral, apoptotic, hypoglycemic, hypolipidemic, immunomodulatory, and memory enhancing properties [1,2,3]. Phytochemical investigations have revealed the presence of several bioactive alkaloids, coumarins, flavonoids, phenolics, polyacetylenes, and terpenes with diverse functionalities and therapeutic attributes. Butyrate is a potent anti-tumor agent because of its ability to induce histone hyperacetylation which further augments cellular differentiation, cell cycle arrest and apoptosis when tested in a variety of cancer cell lines [4]. In addition to this, the anti-cancer effect of linoleic acid have been demonstrated against human breast, colon, and colorectal carcinomas and mice colon, epidermal, hepatic, mammary, prostate, and stomach carcinomas [5]. The role of oleic acid in the chemoprevention of human breast, gastric, and tongue squamous cell carcinomas is illustrated in the cell culture-based assays by Li et al. [6] and Jiang et al. [7]. Other fatty acids like myristic acid provides chemoprotection against breast cancer [8], margaric acid worked against lung cancer [9], and palmitic acid has been shown to be effective against colon and colorectal cancers [10,11]. Previous scientific reports were manually integrated to anticipate the anti-cancer potential of *Foeniculum vulgare* phytoconstituents against different types of cancers, and these included anisaldehyde [12], γ-asarone [13], carvone [14], chlorogenic acid [15], estragole [16], eugenol [17], fenchone [18], γ-terpinene [19], D-limonene [20], myrcene [21], α-pinene [22], quercetin-3-O-beta-D-glucuronide [23], tarns-anethole [24], α-terpineol [25], and vinylguaiacol [26], as indicated in Table 1.

Assays have demonstrated the anti-cancer potential of some important *Foeniculum vulgare* constituents against a wide array of cancers including human breast cancer. Using In silico approaches, we can simultaneously screen out millions of phytocompounds and drugs against any diseases. Furthermore, molecular dynamics-based screening helps to hypothesize the efficacy, stability and toxicity of the important drug candidates or pytocompounds and to design experiments for their in vivo testing. Therefore, the present study was undertaken to propose potential cytotoxic phytoconstituents of *Foeniculum vulgare* using in silico approaches for developing novel therapeutics for the management of human breast cancer. An important drug target candidate in the case of human breast cancer is an Estrogen Receptor α Y537S protein (PDB ID 6CHZ) which was selected on the basis of previous literature.

## 2. Results

### 2.1. Molecular Docking of Major Phytoconstituents of Foeniculum vulgare with Breast Cancer Target 

Forty phytoconstituents were selected for the molecular docking studies with the Estrogen Receptor α Y537S breast cancer target protein (PDB ID 6CHZ).

**Figure 1:**
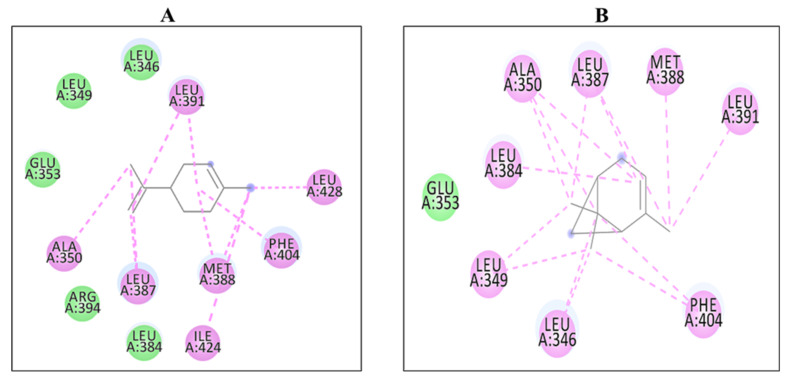
2D Interaction complexes of (**A**) D-limonene and (**B**) α-pinene with 6CHZ protein.

**Figure 2:**
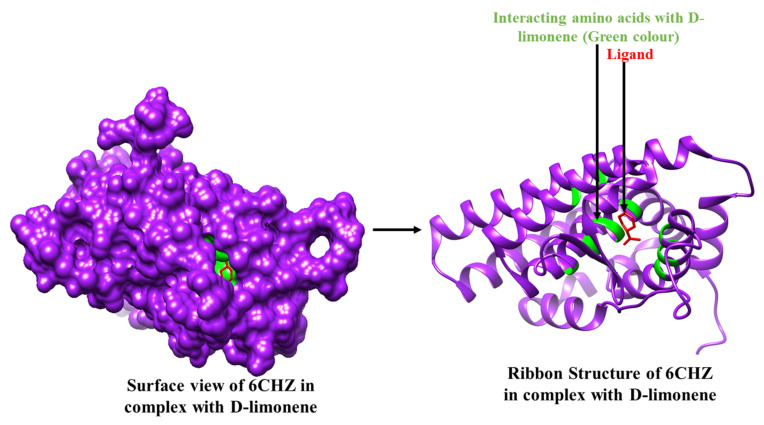
3D Interaction complex of α-pinene with 6CHZ protein; where purple shows the target protein, green shows the hydrophobic interactions, and red shows the ligand molecule.

**Figure 3:**
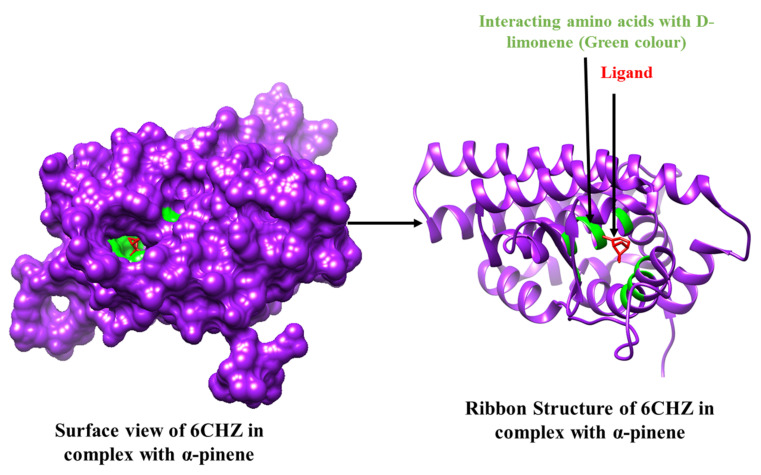
3D Interaction complex of D-limonene with 6CHZ protein; purple shows the target protein, green shows the hydrophobic interactions, and red colour is shows the ligand molecule.

Among all of the selected phytoconstituents, α-pinene and D-limonene showed best interactions with 6CHZ with binding energies of −6.0 and −5.9 kcal/mol, respectively, followed by 1-(1-cyanocyclohexyl) pyrrolidine, α -terpineol and piperitinone (with interaction energy of −5.8 kcal/mol), eugenol, and carvone (with interaction energy of −5.6 kcal/mol), camphor, gamma-terpinene, linoleic acid, and trans-anethole (with interaction energy of −5.5 kcal/mol), α-d-glucose, and chlorogenic acid (with interaction energy of −5.3 kcal/mol), fenchone, gamma-asarone, methyl-chavicole, methyl benzaldehyde, and vinyl guaiacol (with interaction energy of −5.2 kcal/mol), quercetin−3-O-beta-D-glucuronide and ascorbic acid (with interaction energy of −5.1 kcal/mol). Other phytoconstituents such as 1,2-dithiocane, acetic acid, anisaldehyde, estragole, margaric acid, mesitol, myrcene, myristic acid, N-valeramide, N-octadecane, oleic acid, palmitic acid, pelargic acid, pentyl vinyl carbinol, petroselinic acid, stearic acid, syringol, and *Z,Z*-heptadeca-8,11-dien-1-yl bromide showed binding energies less than 5 kcal/mol (Table 2). Alpha-pinene showed hydrophobic interactions with Leu 346, Leu 349, Ala 350, Glu 353, Leu 384, Leu 387, Met 388, Leu 391, and Phe 404 and D-limonene with Leu 346, Leu 349, Ala 350, Glu 353, Leu 384, Leu 387, Met 388, Leu 391, Arg 394, Phe 404, Ile 424, and Leu 428 residues of the cancer drug target protein 6CHZ, as shown in Table 2. 2D interactions of the most stable protein-ligand complexes are shown in Figure 1 and 3D interactions are shown in Figure 2 and Figure 3.

### 2.2. Drug Likeness Prediction of Foeniculum vulgare Phytoconstituents 

The drug likeness parameters of all the selected phytoconstituents were assessed using Molinspiration web server. Among all the selected phytocompounds, acetic acid, anisaldehyde, ascorbic acid, gamma-asarone, trans-anethole, benzeneethanamine, camphor, carvone, 1-cyanocychexylpyrrolidin, 1-2-dithiocane, estragole, eugenol, fenchone, alpha-d-glucose, D-limonene, methylchavicole, myrcene, 1-octen-3-ol, pelargic acid, alpha-pinene, piperitinone oxide, syringol, alpha-terpeneol, gamma-terpinene, p-tolualdehyde, and N-valeramide, passed different criteria of drug likeness and can be used as future oral drugs (Table 3). On the basis of molecular docking and toxicity assessments, alpha-pinene and D-limonene both were found to be the most suitable drug candidates and hence, were selected for MD simulation studies to verify the stability of protein-ligand complexes.

### 2.3. Toxicity Prediction of Foeniculum vulgare Phytoconstituents 

The toxicity of the selected forty phytoconstituents was analyzed by using the Protox II online tool. Toxicity data showed that among all the selected phytoconstituents, anisaldehyde, gamma-asarone, trans-anethole, estragole, p-tolualdehyde, and quercetin-3-O-beta-D-glucuronide are carcinogenic in nature, alpha-d-glucose and octadecenoic acid are hepatotoxic in nature, alpha-d-glucose, and quercetin-3-O-beta-D-glucuronide are immunotoxic in nature, and gamma-asarone is mutagenic in nature (Table 4). Molecular docking and toxicity data revealed that alpha-pinene and D-limonene are the best drug molecules for the management of human breast cancer. Alpha-pinene belongs to class 5 drugs with an LD_50_ of 3700 mg/kg body weight and D-limonene is a class 4 drug with an LD_50_ of 500 mg/kg body weight. The detailed in silico toxicity parameters are depicted in Table 4.

### 2.4. Molecular Dynamic simulation of Complexes

**Figure 4:**
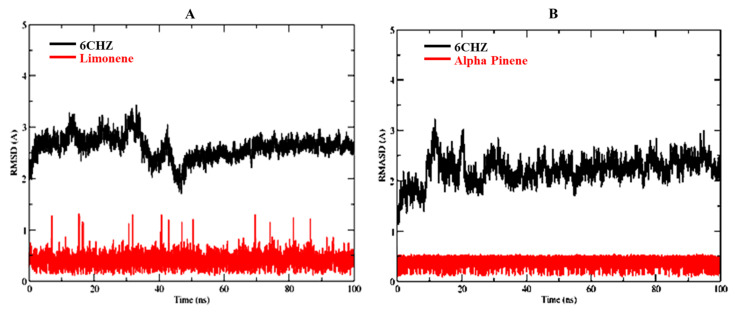
RMSDs of protein-ligand complexes: (**A**) D-limonene with 6CHZ and (**B**) Alpha-pinene with 6CHZ. Red indicates ligand and black indicates C-alpha of the target protein.

**Figure 5:**
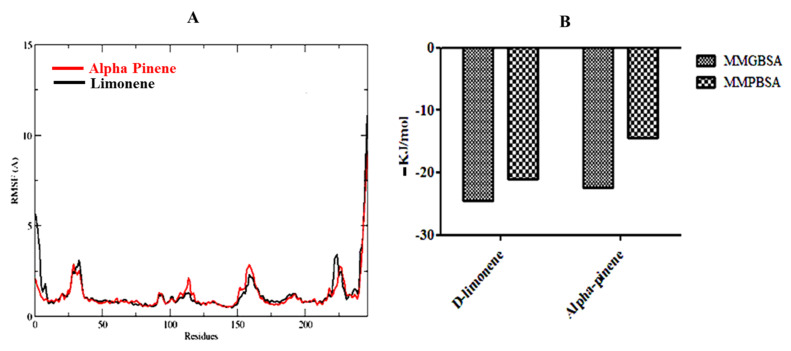
(**A**) RMSF plots of D-limonene and alpha-pinene complexes with 6CHZ (red is indicating D-limonene and black is indicating alpha pinene) and (**B**) MMGBSA and MMPBSA plots of D-limonene and alpha-pinene complexes with 6CHZ.

Based on molecular docking, and toxicity investigations, alpha-pinene and D-limonene were found to be the choicest drug candidates for managing human breast cancer. Therefore, alpha-pinene and D-limonene ligand complexes with breast cancer target protein 6CHZ were selected for MD simulation studies to further verify stability of these complexes. The RMSDs of D-limonene complex with 6CHZ C-alpha was stable from the start of simulation up to 40 ns (3 Å). It showed little fluctuation between 40–50 ns (which is in the acceptable range), and afterwards it remained stable up to 100 ns at 2.9 Å (Figure 4A). Whereas, in the case of 6CHZ complex with alpha-pinene, RMSDs of C-alpha showed little fluctuation between 0–30 ns (3 Å), which remained stable between 30 to 100 ns thereafter at 2.7 Å (Figure 4B). The RMSF plots of D-limonene and alpha-pinene fitted over 6CHZ protein also showed lesser residual fluctuations in the protein’s secondary structures, such as the alpha helices and beta strands (Figure 5A).

The MMGBSA (Molecular Mechanics/Generalized Born Surface Area) of alpha-pinene is −22.39 and for D-limonene, it is −24.52 kJ/mol; whereas, the MMPBSA of alpha-pinene is −14.44 and D-limonene is −21.08 kJ/mol (Figure 5).

**Figure 6:**
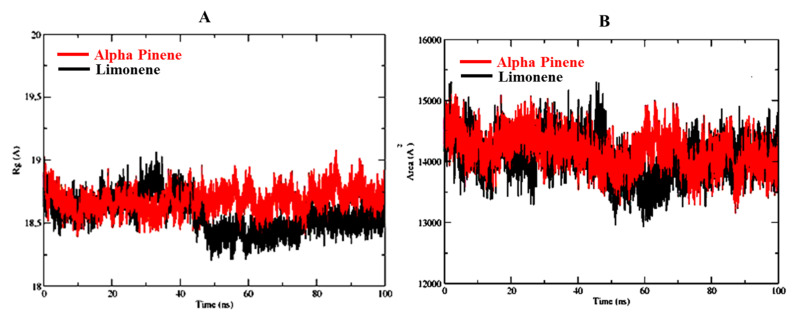
(**A**) Radius of gyration (ROG) and (**B**) Solvent Accessible Surface Area of the best protein-ligand complexes. Red is indicating D-limonene and black is indicating α-pinene.

Radius of gyration of α-pinene and D-limonene in complex with 6CHZ is in the range of 18–19 Å and as shown in Figure 6A. The radius of gyration plots establishes the compactness of the α-pinene and D-limonene in complex with 6CHZ protein complex and confirms the stability of complexes. Solvent accessible range of alpha pinene and D-limonene complexed with 6CHZ protein is between 14,000–15,000 Å; as shown in Figure 6B.

## 3. Discussion

Medicinal herbs always confer beneficial effects on human health when consumed in moderate quantities. *Foeniculum vulgare*, especially being rich in several bioactive constituents, has been used as a food condiment and ingredient throughout the world. It has been traditionally used since ancient times to cure several human diseases including arthritis, cancers, conjunctivitis, endocrine, gastric, hepatic, insomnia, kidney, reproductive, and respiratory ailments. These studies signify that *F. vulgare* holds a promising future, and harnessing its hidden anti-cancer potential could be an important milestone in the field of novel drug development. However, the development of potent cytotoxic agents requires investigation of the molecular mechanisms of disease prevention to substantiate the beneficial attributes as well as to authenticate the immense pharmacological importance of *Foeniculum vulgare* constituents. 

In the current study we found that among all forty phytocompounds, α- pinene and D-limonene showed the best binding affinity with the breast cancer target. They were also found to be non-toxic in nature. In contrast to our study, Ghasemian et al. [35] reported the anticancer activity of *F. vulgare* against the MCF-7 breast cancer cell line by MTT assay. They also reported that the essential oil *F. vulgare* increases the expression of *Bax* and decreases the *Bcl2* gene expression. Similarly, Mohamad et al. [36] reported the anticancer activity of *F. vulgare* methanolic extract and essential oil against the MCF-7 breast cancer cell line and Hepg-2 liver cell line by in vivo and in vitro assays. Berrington and Lall [37] also reported the anticancer activity of *F. vulgare* acetone extract against the Vero African green monkey kidney cell line and the cervical cancer cell line HeLa. Batool et al. [38] also reported the anticancer activity of *F. vulgare* methanolic and ethanolic extract against the MCF-7 breast cancer cell line by MTT assay. Both extracts are more effective against the breast cancer cell line (methanol and ethanol—40 µg/mL). Zaahkouk et al. [39] also reported the anticancer activity of methanolic extract of *Foeniculum vulgare* seed against breast, colon and liver cancer. Similar to our study, Hossain [40] studied the anticancer activity of *Withania somnifera* phytocompounds with 6CHZ protein by molecular docking. He also reported the drug likeness and ADMET activity of phytocom pounds. There are several reports on molecular docking, MD simulation, and ADMET screening of phytoconstituents [41,42,43,44,45,46]. 

Phytocompounds of *Rheum emodi* were screened for antibacterial and antiviral properties by molecular docking, and results were validated by MD simulation [41]. Similarly, Salaria et al. [42,43] studied the antimicrobial activity of *T. serpyllum* compounds and the antioxidant and anti-inflammatory activities of important phytoconstituents were investigated using molecular docking and MD simulation, and the results were further validated by in vitro experiments. In addition, phytocompounds containing nanoparticles were shown to provide significant anti-cancer effects against breast cancer cell lines (MCF-7, PC-12, MDA-MB-231) [47,48,49,50,51]. Letrozole incorporated folate-conjugated polymer-lipid hybrid nanoparticles were also shown to exhibit anticancer activity against the MCF-7 and PC-12 cell lines [52]. Folic acid functionalized apoferritin is a drug delivery vehicle for equirubicin against breast cancer cells (MCF-7) [53].

## 4. Materials and Methods

### 4.1. Bioinformatics Tools

Open Babel GUI (O’Boyle et al., 2011), UCSF Chimera 1.8.1, PubChem (www.pubchem.com, accessed on 15 November 2021), RCSB PDB (http://www.rscb.org/pdb, accessed on 20 December 2021), AutoDock/vina software (https://vina.scripps.edu, accessed on 1 May 2022) [54], and Discovery Studio were used in the present investigation.

### 4.2. Ligand Preparation

40 major phytoconstituents found in the medicinal plant *Foeniculum vulgare* Mill were selected on the basis of the literature and were further selected for molecular docking studies. The three-dimensional structures of the selected phytoconstituents were downloaded from the PubChem database (www.pubchem.com, accessed on 15 November 2021) in .sdf formats. The .sdf files of the phytoconstituents were converted into PDB formats. Open Babel software was used to prepare all of the selected ligands (phytoconstituents) from the command line on an Ubuntu terminal. Chemdraw 3D version 15.0 (PerkinElmer, Waltham, MA, USA) was used to reduce the energy of all of the identified phytocompounds. 

### 4.3. Protein Preparation 

Estrogen Receptor α Y537S breast cancer target (PDB ID 6CHZ) was used for molecular docking with major phytocompounds from *Foeniculum vulgare* Mill. The three-dimensional 6CHZ was downloaded from the protein databank (http://www.rscb.org/pdb, accessed on 20 December 2021). It consists of a dimeric structure, and its chain A was extracted for docking using Pymol software. The active site was predicted manually by grid box analysis (grid dimensions x = 72, y = 74, z = 76 Å, center at x, y, z = −28.959, −2.617, −25.683 Å). 

### 4.4. Molecular Docking of Major Phytoconstituents of Foeniculum vulgare with Breast Cancer Target 

The selected ligands were docked to the catalytic triad of proteins using AutoDock vina, which was then saved as a pdbqt file. The population of potential ligand conformations/orientations at the binding site was estimated via docking. A vina script was used to align the ligands in the same spatial coordinates [54]. After the docking search was completed, the best conformation with the lowest docked energy was chosen. Discovery Studio (https://discover.3ds.com/d, accessed on 20 December 2021) was used to study interactions between proteins and ligands in the pdb complex preparations. A negative score (kcal/mol) was used to calculate the ligand’s binding strength. The equilibrium constant was calculated by using formula [55]:

Ki = e^−ΔG/RT^ ΔG = Gibbs free energy; R = 1.9872 cal/mol·K; T = 298.15 °K

### 4.5. Drug Likeness Prediction of Foeniculum vulgare Phytoconstituents

Drug likeness prediction of *F. vulgare* phytoconstituents was done using the Molinspiration online server (http://www.molinspiration.com, accessed on 20 December 2021). Drug likeness is based on the Lipinski’s rule of five. According to the rule of five, the number of hydrogen acceptors should be <10, the number of hydrogen donors should be <5, the molecular weight should be <500 Da, and the partition coefficient should be >5 (estimated in terms of log P) in acceptable drug molecules. In the case of variables, only one violation is acceptable [56].

### 4.6. Toxicity Prediction of F. vulgare Phytoconstituents by Protox II Server 

The pharmacokinetics and toxicity of pharmacologically important phytoconstituents were predicted by using Protox II servers. Toxicity was estimated in terms of LD50 values ranging from ≤50 mg/kg (in the case of Class I compounds), between 50 to 500 mg/kg (in the case of Class II compounds), between 500 to 5000 mg/kg (in the case of Class III compounds), and >5000 mg/kg (in the case of Class IV compounds). Classes I, II, and III have less toxicity, whereas Class IV displays no toxicity [57]. Moreover, PROTOX is a rodent oral toxicity server that is used to determine LD50 values and toxicity classes of potentially cytotoxic agents [58]. Based on the molecular docking drug-likeness data and toxicity data, phytoconstituents were selected for further MD simulation analysis.

### 4.7. MD Simulation of Best Protein-Ligands Complexes

MD simulations of best protein-ligand complexes were done by using the Amber18 tool. MD simulations were performed to gain a better understanding of the binding interactions of the 6CHZ protein with the selected phytoconstituents, namely α-pinene and D-Limonene [51]. The ligands underwent an amber generalized force field (GAFF), while the protein was subjected to amber ff14SB [52,53]. Using Gaussian 09 software, the atomic charges of the ligands were computed using the restrained electrostatic potential (RESP) procedure at the HF/6-31G* level of theory 31, 32 [55]. Using an H++ server, proton transfer states of the ionizable residues in protein structures were investigated using the pKa method at a neutral pH. The tLeap application was used to create each system. Each system was solvated in a cubic water box with the TIP3P model after being treated with sodium ions. Each system was exposed to at least four minimizations in order to eliminate the worst conflicts. Initially, all of the sodium ions and water molecules were reduced using a steepest descent technique of 2000 steps, followed by a conjugate gradient algorithm of 3000 steps. The same approach was used to relax all of the hydrogen and water molecules in a row. Finally, the entire system was energy-minimized for 5000 steps of steepest descent and 5000 steps of conjugate gradients. The system was heated from 0 to 300 K while performing 200 ps of MD and then 300 ps of density equilibration at a fixed volume with position restrictions on the protein atoms. All protein-ligand complexes were stabilized with for 10 ns of MD without any structural restrictions at a constant pressure before the manufacturing process. By linking to a Langevin thermostat with a collision frequency of 2 cm^−1^, the temperature was kept at 300 K. For the unpaired electron interactions, a cut off of 10 was chosen, and the Particle Mesh Ewald (PME) [53] approach and the SHAKE algorithm was used to limit the bond lengths involving hydrogen atoms. Finally, at a temperature of 300 K, MD simulations (productions) were run for 100 ns. The computed trajectories were utilized to examine activities of all the complexes in order to determine the stability of the system. Important parameters like root mean square deviation (RMSD), root mean square fluctuation (RMSF), radius of gyration (RG), and solvent accessible surface area (SASA) were used to examine deviations of the protein and protein-ligand complexes [51,52]. Furthermore, using molecular mechanics and the Poisson–Boltzmann Surface Area (MM-PBSA) method, the total free energy of binding, the free energy of solvation (polar vs. non-polar solvation energies), and the potential energy (electrostatic and van der Waal’s interactions) of each protein-ligand complex were calculated. For the MM-PBSA computation, the last 10 ns of the MD trajectory were used [53].

## 5. Conclusions

Forty major phytoconstituents of *Foeniculum vulgare* were screened for breast cancer by molecular docking with the 6CHZ target protein. Among all of the selected phytoconstituents, D-limonene and α-pinene have the best binding affinity and follow all the parameters of toxicity. An MD simulation study validated the stability of complexes. α-pinene has a lot of potential for the treatment of breast cancer, and this hypothesis can be further validated by in vitro and in vivo experiments. 

## Figures and Tables

**Table 1:** Anti-cancer bioactive phytoconstituents of *Foeniculum vulgare* Mill.

Sr. No.	Phytoconstituent (Scientific Name)	Targeted Cancer Cell Lines
	1-(1-Cyanocyclohexyl) pyrrolidine	-
1.	1,2-Dithiocane (1,2-dithiacyclooctane)	-
2.	Acetic acid	Gastric (RGM1, RGK1, RGM-GFP and RGK-KO) cells [27]
3.	Anisaldehyde (4-methoxybenzaldehyde)	Breast (MCF-7), epidermoid carcinoma (ME 180), liver (HepG2) cells [12]
4.	Ascorbic acid	Bladder, breast, cervical, colorectal, esophageal, leukemia, lung, non-Hodgkin’s lymphoma, pancreatic, prostate, salivary gland, and stomach cancers [28]
5.	Camphor	Mouse model of keratinocyte-derived skin cancer [29]
6.	Carvone	Human melanoma (A375) and breast (MDA-MB468) cells [14] neuroblastoma (N2a) cells [22]
7.	Chlorogenic acid	Colon, glioma, hepatic, lung cancers (Human A549-5FU, Bel-7402, CCC-HEL-1, HCT-116, HEK293T, HH, Huh7, iPS, M059J, MIHA, MRC-5, NCI-H358, NCI-H446, SK- LU-1, U87MG, WI-38; rat C6; mouse G422 cells) [15]
8.	D-limonene	Human breast, colorectal, hepatic, epithelial cell carcinomas; rat and mice liver cancer, pulmonary adenoma, forestomach tumors [20]
9.	Eicosamethyl-cyclodecasiloxane (Icosamethyl-cyclodecasiloxane)	Breast (MCF-7), ovary (A2780), colon (HT29) cells [30]
10.	Estragole (Methyl Chavinol)	Breast (MCF-7) cells [16,31]
11.	Eugenol	Breast, cervical, colon, colorectal, gastric, leukemia, lung, prostate and skin cancers [17]
12.	Fenchone	Ehrlich ascites carcinoma [18]
13.	Iron monocarbonyl- (1,3-butadiene-1,4-dicarbonic acid, diethyl ester)	-
14.	Linoleic acid (cis-9,cis-12-Octadecadienoic acid)	Human breast, colon, colorectal carcinomas; mice colon, epidermal, hepatic, mammary, prostate and stomach carcinomas [5]
15.	Margaric acid (Heptadecanoic acid)	Lung (PC-9 NSCLC) cells [9]
16.	Mesitol (2,3,5-trimethyl phenol)	-
17.	Methyl benzaldehyde (*p*-tolualdehyde)	-
18.	Myrcene	Lung (A549) cells [21]
19.	Myristic acid (Tetradecanoic acid)	Breast cancer [21]
20.	N-valeramide	-
21.	Octadecane	-
22.	Oleic acid (cis-9-octadecenoic acid)	Human breast (MDA-MB-231), gastric (HGC-27) cells; tongue squamous cell carcinoma (UM1 and CAL27)) [6,7]
23.	Palmitic acid (Hexadecanoic acid)	Colon (HT29), colorectal (HCT-116) [10, 11]
24.	Pelargic acid (1-Octanecarboxylic acid, Nonanoic acid)	-
25.	Pentyl vinyl carbinol (1-Octen-3-ol)	-
26.	Petroselinic acid (6-Octadecylenic acid)	-
27.	Phenyethylamine (Benzene ethanamine)	Breast (MCF-7) cells [32]
28.	Piperitinone oxide	-
29.	Quercetin-3-o-beta-d-glucuronide	Breast (MDA-MB-231) cells [23]
30.	Stearic acid (octadecanoic acid)	Breast (MDA-MB-361, MCF-7, MDA-MB-231) cells [33]
31.	Syringol (2,6-dimethoxyphenol)	-
32.	Trans-anethole	Breast (MCF-7) cells; Oral (Ca9-22) [24,31,34]
33.	Vinylguaiacol (2-methoxy-4-vinylphenol)	Human pancreatic (Panc-1 and SNU-213) cells [26]
34.	*Z,Z*-heptadeca-8,11-dien-1-yl bromide	-
35.	α-d-glucose	-
36.	α-Pinene	Neuroblastoma (N2a) cells [22].
37.	α-terpineol	Breast (MCF-7), cervix (Hela), colorectal (HCT-8, SW620, HCT-116, HT-29), leukemia (CCRF-CEM), lymphoma (U937 GTB), myeloma (RPMI 8226), renal adenocarcinoma (drug resistant 8226/Dox40, 8226/LR5, CEMVM-1, U937-vcr, H69AR and the primary resistant ACHN) and small cell lung cancer (NCI-H69) cells [25]
38.	γ-asarone	Gastric (AGS) cells [13]
39.	γ-terpinene	Human prostate (LNCaP, PC-3), glioblastoma (SF-763, SF-767) cells [19]

**Table 2:** Binding energies of important *Foeniculum vulgare* constituents with breast cancer target and the interacting amino acids.

Common Phytoconstituents (Scientific Name)	Energy (kcal/mol)	Inhibition Constant (K_i_)	H-Bonding	Interacting Amino Acids
1,2-Dithiocane (1,2-dithiacyclooctane)	−4.7	1.0 × 10^15^	Asp 537, Asp 538, Leu 544, Asp 545	Leu 372, Val 376, Leu 539, Leu 540, Glu 542, Met 543
1-(1-Cyanocyclohexyl) pyrrolidine	−5.8	1.0 × 10^15^	-	Leu 346, Leu 349, Ala 350, Glu 353, Leu 387, Met 388, Leu 391, Arg 394, Phe 404
Acetic acid	−3.4	1.0 × 10^15^	Asn 455, Ser 456, Leu 511, Arg 515	Glu 385, Ile 451, Ile 452, Ser 512
Anisaldehyde (4-methoxy benzaldehyde)	−4.9	1.0 × 10^15^	Arg 394	Leu 346, Thr 347, Leu 349, Ala 350, Glu 353, Leu 384, Leu 387, Leu 391, Phe 404
Ascorbic acid	−5.1	1.0 × 10^15^	Glu 380, Ser 381, Ser 456, Ser 518	Trp 383, Glu 385, Asn 519, Met 522
Camphor	−5.5	1.0 × 10^15^	-	Leu 349, leu 346, leu 391, Ala 350, Leu 384, Met 388, Leu 387, Trp 383
Carvone	−5.6	1.0 × 10^15^	-	Leu 248, Ile 424, Leu 346, Ala 350, Phe 404, Met 388, Leu 384, Leu 391, Leu 349, Arg 394, Leu 387, Glu 353
Chlorogenic acid	−5.3	1.0 × 10^15^	Ser 381, Thr 460, Ser 456, Asn 519	Glu 523, Tyr 526, Met 522, His 377, Gly 457
D-Limonene	−5.9	1.0 × 10^15^	-	Leu 346, Leu 349, Ala 350, Glu 353, Leu 384, Leu 387, Met 388, Leu 391, Arg 394, Phe 404, Ile 424, Leu 428
Eicosamethyl-cyclodecasiloxane (Icosamethyl-cyclodecasiloxane)	N/A	1.0 × 10^15^	-	-
Estragole	−4.9	1.0 × 10^15^	Thr 347	Met 343, Leu 346, Ala 350, Trp 383, Leu 384, Leu 387, Leu 525
Eugenol	−5.6	1.0 × 10^15^	-	Leu 428, Leu 387, Arg 394, Glu 353, Met 388, Ala 350, Leu 346, Leu 391, Leu 349, Ile 424, Phe 404
Fenchone	−5.2	1.0 × 10^15^	-	Leu 346, Ala 350, Glu 353 Leu 387, Met 388, Arg 394, Phe 404
Linoleic acid (cis-9,cis-12-Octadecadienoic acid)	−5.5	1.0 × 10^15^	-	Met 343, Leu 346, Leu 349, Ala 350, Met 383, Leu 384, Leu 387, Met 388, Leu 391, Arg 394, Phe 404, Met 421, Ile 424, Leu 428, Gly 521, His 524, Leu 525
Margaric acid (Heptadecanoic acid)	−3.6	1.0 × 10^15^	Asp 332, Glu 339	Glu 330, Tyr 331, Arg 335, Pro 336, Ala 340, Ser 341, Gly 344, Asn 348
Mesitol (2,3,5-trimethyl phenol)	−4.7	1.0 × 10^15^	-	Leu 346, Leu 349, Leu 387, Glu 353, Met 522, Leu 526
Methyl-chavicole	−5.2	1.0 × 10^15^	-	Phe 404, Leu 391, Leu 349, Leu 525, Arg 394, Leu 387, Glu 353, Ala 350, Leu 346, Trp 383, Leu 384, Leu 525
Methyl benzaldehyde (p-tolualdehyde)	−5.2	1.0 × 10^15^	Arg 394	Leu 346, Leu 349, Ala 350, Glu 353, Leu 387, Met 388, Leu 391, Phe 404
Myrcene	−4.7	1.0 × 10^15^	-	Ala 350, Leu 354, Trp 383, Leu 536, Asp 351, Met 522, Leu 525, Tyr 526
Myristic acid (Tetradecanoic acid)	−4.0	1.0 × 10^15^	Val 533	Asn 532, Val 534, Pro 535, Leu 354, Tyr 526, Cys 530, Met 522, Leu 536, Leu 526, Trp383
N-Valeramide	−4.2	1.0 × 10^15^	Arg 394	Met 343, Leu 346, Ala 350, Leu 391, Phe 404
Octadecane	−3.8	1.0 × 10^15^	-	Trp 383, Met 522, Leu 525, Tyr 526, Lys 529, Cys 530, Val 533, Leu 536
Oleic acid (cis-9-octadecenoic acid)	−4.2	1.0 × 10^15^	-	-
Palmitic acid (Hexadecanoic acid)	−4.7	1.0 × 10^15^	Ser 381, Ser 456, Thr 460	Glu 380, Gly 457, Glu 523, Met 522, Tyr 526
Pelargic acid (1-Octanecarboxylic acid, Nonanoic acid)	−4.1	1.0 × 10^15^	Ser 381, Thr 460, Arg 515	Ser 456, Gly 457, Ser 518, Asn 519, Met 522
Pentyl vinyl carbinol (1-Octen-3-ol)	−4.7	1.0 × 10^15^	-	Leu 349, Ala 350, Leu 346, Leu 387, Phe 404, Glu 353, Met 388, Leu 391
Petroselinic acid (6-Octadecylenic acid)	−4.2	1.0 × 10^15^	-	Arg 515, Ser 518, Met 522, Ser 381, Asn 519, Glu 380, Thr 460, His 377, Glu 523
Phenyethylamine (Benzene ethanamine)	−4.7	1.0 × 10^15^	-	Leu 346, Thr 347, Leu 349, Ala 350, Glu 353, Leu 384, Leu 387, Met 388, Leu 391, Arg 394, Phe 404, Leu 525
Piperitinone oxide	−5.8	1.0 × 10^15^	-	Leu 346, Leu 349, Ala 350, Trp 383, Leu 384, Leu 387, Met 388, Leu 391, Arg 394, Phe 404, Ile 424, Leu 428
Quercetin-3-o-beta-d-glucuronide	−5.1	1.0 × 10^15^	Tyr 526, Cys 530	Pro 535, Leu 525, Lys 529, Lys 531, Val 533, Asn 532, Lys 531, Met 522, Trp 383, Glu 380, Ser 537, Pro 535
Stearic acid (octadecanoic acid)	−4.7	1.0 × 10^15^	-	Arg 394, Glu 353, Leu 525, Trp 383, Leu 535, Leu 391, Leu 387, Leu 384, Ala 350, Thr 347, Leu 354, Asp 351, Leu 539, Phe 404, Met 388, Leu 346
Syringol (2,6-dimethoxy phenol)	−4.7	1.0 × 10^15^	-	Leu 346, Leu 349, Ala 350, Glu 353, Leu 384, Leu 387, Met 388, Leu 391, Phe 404, Leu 428
Trans-anethole	−5.5	1.0 × 10^15^	-	Leu 346, Leu 349, Ala 350, Glu 353, Leu 387, Leu 391Arg 394, Phe 404, Met 434, Leu 525
Vinyl guaiacol (2-methoxy-4-vinylphenol)	−5.2	1.0 × 10^15^	-	Leu 346, Leu 349, Ala 350, Glu 353, Leu 387, Met 388, Leu 391, Arg 394, Phe 404, Leu 428
Z,Z-heptadeca-8,11-dien-1-yl bromide	−3.7	1.0 × 10^15^	-	Asn 519, Glu 523, Met 522, Ser 381, Tyr 526
α-d-Glucose	−5.3	1.0 × 10^15^	Glu 380, Ser 381, Arg 515, Asn 519	His 377, Ser 456, Gly 457, Thr 460, Ser 518, Met 522
α-Pinene	−6.0	1.0 × 10^15^	-	Leu 346, Leu 349, Ala 350, Glu 353, Leu 384, Leu 387, Met 388, Leu 391, Phe 404
α-terpeneol	−5.8	1.0 × 10^15^	-	Trp 383, Thr 347, Leu 525, Leu 346, Leu 384, Leu 387, Ala 350, Leu 349, Phe 404, Glu 353, Leu 391
γ-asarone	−5.2	1.0 × 10^15^	-	Glu 380, Ser 381, Trp 383, Tyr 526, Met 522, Ser 381, Glu 523, Asn 519, Ser 518
γ-terpinene	−5.5	1.0 × 10^15^	-	Ala 350, Leu 349, Glu 353, Arg 394, Phe 404, Leu 428, Met 421, Leu 391, Met 388, Leu 387, Ile 424, Leu 346, Leu 384

Best phytococonsituents are highlited with grey colour.

**Table 3:** Drug-likeness prediction of *Foeniculum vulgare* phytoconstituents using the Molinspiration web server.

Phytocompounds	Log P	Polar Surface Area (Å^2^)	No. of atoms	No. of Nitrogen and Oxygen	No. of -OH and -NHn	Violations	Number of rotations	MW
1-2-Dithiocane	2.75	0.00	8	0	0	0	0	148.30
1-Cyanocychexylpyrrolidin	−0.48	44.10	10	3	0	0	1	138.17
Acetic acid	−0.23	37.30	4	2	1	0	0	60.05
Anisaldehyde	1.78	26.30	10	2	0	0	2	136.15
Ascorbic acid	−1.40	107.22	12	6	4	0	2	176.12
Benzeneethanamine	0.92	26.02	9	1	2	0	2	121.18
Camphor	2.16	17.07	11	1	0	0	0	152.24
Carvone	2.51	17.07	11	1	0	0	1	150.22
Chlorogenic acid	−0.45	164.74	25	9	6	1	5	354.31
D-limonene	3.62	0.00	10	0	0	0	1	136.24
Eicosamethyl-cyclodecasiloxane	3.66	92.34	40	10	0	1	0	741.55
Estragole	2.82	9.23	11	1	0	0	3	148.21
Eugenol	2.10	29.46	12	2	1	0	3	164.20
Fenchone	2.16	17.07	11	1	0	0	0	152.24
Heptadecanoic acid	7.56	37.30	19	2	2	1	15	270.46
Hexadecanoic acid	7.06	37.30	18	2	1	1	14	256.43
Linoleic acid	6.86	37.30	20	2	1	1	14	280.45
Methylchavicole	2.82	9.23	11	1	0	0	3	148.21
Myrcene	3.99	0.00	10	0	0	0	4	136.24
Myristic acid	6.05	37.30	16	2	1	1	12	228.38
Octadecenoic acid	7.82	37.30	20	2	1	1	15	282.47
Pelargic acid	3.52	37.30	11	2	1	0	7	158.24
Petroselinic acid	7.58	37.30	20	2	1	1	15	282.47
Piperitinone oxide	1.76	29.60	122	0	0	0	0	166.22
p-Tolualdehyde	2.18	17.07	9	1	0	0	1	120.15
Quercetin-3-O-beta-D-glucuronide	−0.49	227.57	34	13	8	2	4	478.36
Stearic acid	8.07	37.30	20	2	1	1	16	284.48
Syringol	1.34	38.70	11	3	1	0	2	154.16
Trans-anethole	3.10	9.23	11	1	0	0	2	148.21
Vinylguaiacol	2.15	29.46	11	2	1	0	2	150.18
Z,Z-Heptadeca-8,11-dien-1-yl bromide	6.93	17.07	18	1	0	1	13	250.43
α-d-Glucose	−2.64	110.37	12	6	5	0	1	180.16
α-Pinene	3.54	0.00	10	0	0	0	0	136.24
α-Terpineol	2.60	20.23	11	1	1	0	1	154.25
Pentyl vinyl carbinol (1-Octen-3-ol)	2.76	20.23	9	1	1	0	5	128.22
γ-Asarone	2.39	27.70	15	3	0	0	5	208.26
γ-Terpinene	3.36	0.00	10	0	0	0	1	136.24

Best phytococonsituents are highlited with grey colour.

**Table 4:** Toxicity assessment of *Foeniculum vulgare* phytoconstituents using Protox II.

Phytocompounds	Protox II					
	LD_50_, (mg/kg)	Hepatotoxicity	Carcinogenicity	Immunotoxicity	Mutagenicity	Cytotoxicity
1-2-Dithiocane	620 (Class 4)	Inactive	Inactive	Inactive	Inactive	Inactive
1-Cyanocychexylpyrrolidin	1650 (Class 4)	Inactive	Inactive	Inactive	Inactive	Inactive
Acetic acid	333 (Class 1)	Inactive	Inactive	Inactive	Inactive	Inactive
Anisaldehyde	1550 (Class 4)	Inactive	Active	Inactive	Inactive	Inactive
Ascorbic acid	3767 (Class 5)	Inactive	Inactive	Inactive	Inactive	Inactive
Benzeneethanamine	400 (Class 4)	Inactive	Inactive	Inactive	Inactive	Inactive
Camphor	775 (Class 4)	Inactive	Inactive	Inactive	Inactive	Inactive
Carvone	1640 (Class 4)	Inactive	Inactive	Inactive	Inactive	Inactive
Chlorogenic acid	5000 (Class 5)	Inactive	Inactive	Active	Inactive	Inactive
D-limonene	500 (Class 4)	Inactive	Inactive	Inactive	Inactive	Inactive
Eicosamethyl-cyclodecasiloxane	1540 (Class 4)	Inactive	Inactive	Inactive	Inactive	Inactive
Estragole	1203 (Class 4)	Inactive	Active	Inactive	Inactive	Inactive
Eugenol	1930 (Class 4)	Inactive	Inactive	Inactive	Inactive	Inactive
Fenchone	775 (Class 4)	Inactive	Inactive	Inactive	Inactive	Inactive
Linoleic acid	10000 (Class 6)	Inactive	Inactive	Inactive	Inactive	Inactive
Margaric acid (Heptadecanoic acid)	900 (Class 4)	Inactive	Inactive	Inactive	Inactive	Inactive
Methyl benzaldehyde (*p*-Tolualdehyde)	1600 (Class 4)	Inactive	Active	Inactive	Inactive	Inactive
Methyl chavicole	1230 (Class 4)	Inactive	Active	Inactive	Inactive	Inactive
Myrcene	5000 (Class 5)	Inactive	Inactive	Inactive	Inactive	Inactive
Myristic acid	900 (Class 4)	Inactive	Inactive	Inactive	Inactive	Inactive
N-valeramide	400 (Class 4)	Inactive	Inactive	Inactive	Inactive	Inactive
Octadecane	750 (Class 3)	Inactive	Inactive	Inactive	Inactive	Inactive
Octadecenoic acid	1925 (Class 4)	Active	Inactive	Inactive	Inactive	Inactive
Pelargic acid	900 (Class 4)	Inactive	Inactive	Inactive	Inactive	Inactive
Pentyl vinyl carbinol (1-octen-3-ol)	340 (Class 4)	Inactive	Inactive	Inactive	Inactive	Inactive
Petroselinic acid	48 (Class 2)	Inactive	Inactive	Inactive	Inactive	Inactive
Piperitinone oxide	2500 (Class 5)	Inactive	Inactive	Inactive	Inactive	Inactive
Quercetin-3-o-beta-d-glucuronide	5000 (Class 5)	Inactive	Active	Active	Inactive	Inactive
Stearic acid	950 (Class 4)	Inactive	Inactive	Inactive	Inactive	Inactive
Syringol	550 (Class 4)	Inactive	Inactive	Inactive	Inactive	Inactive
Trans-anethole	150 (Class 3)	Inactive	Active	Inactive	Inactive	Inactive
Vinylguaiacol	1560 (Class 4)	Inactive	Inactive	Active	Inactive	Inactive
*Z*,*Z*-Heptadeca-8,11-dien-1-yl bromide	5000 (Class 5)	Inactive	Inactive	Inactive	Inactive	Inactive
α-d-Glucose	1190 (Class 4)	Active	Inactive	Active	Inactive	Inactive
α-Pinene	3700 (Class 5)	Inactive	Inactive	Inactive	Inactive	Inactive
α-Terpeneol	2830 (Class 5)	Inactive	Inactive	Inactive	Inactive	Inactive
γ-Asarone	1000 (Class 4)	Inactive	Active	Inactive	Active	Inactive
γ-Terpinene	2500 (Class 5)	Inactive	Inactive	Inactive	Inactive	Inactive

Best phytococonsituents are highlited with grey colour.

## Data Availability

Not applicable.

## Sample Availability

Yes: D-limonene is available with Rajan Rolta and Deeksha Salaria.
